# Cost-effectiveness of edaravone dexborneol versus dl-3-n-butylphthalide for the treatment of acute ischemic stroke: a Chinese health care perspective

**DOI:** 10.1186/s12889-024-17959-3

**Published:** 2024-02-12

**Authors:** Jianchun Li, Weihan Cao, Fei Zhao, Pengfei Jin

**Affiliations:** 1 Department of Pharmacy, Beijing Hospital, National Center of Gerontology, Institute of Geriatric Medicine，Chinese Academy of Medical Sciences, Beijing Key Laboratory of Assessment of Clinical Drugs Risk and Individual Application (Beijing Hospital), Beijing, China; 2 Department of Pharmacy Administration and Clinical Pharmacy, Pharmaceutical Science, Peking University, Beijing, China; 3 No. 1 Dahua Road, Dongcheng district, Beijing, 100730 P.R. China

**Keywords:** Cost-effectiveness, Acute ischemic stroke, Edaravone dexborneol, Dl-3-n-butylphthalide, Matching-adjusted indirect comparison

## Abstract

**Background:**

Edaravone dexborneol and dl-3-n-butylphthalide are two innovative brain cytoprotective drugs from China that have been approved and widely prescribed for acute ischemic stroke, and the cost of the two drugs are partially paid by the Chinese medical insurance system. This study aimed to investigate and compare the cost-effectiveness of edaravone dexborneol versus dl-3-n-butylphthalide for acute ischemic stroke from the Chinese healthcare system’s perspective.

**Methods:**

A model combining a short-term decision tree model with 90 days and a long-term Markov model with a life-time horizon (40 years) was developed to simulate the cost-effectiveness of edaravone dexborneol versus dl-3-n-butylphthalide for acute ischemic stroke over a lifetime horizon. Since the absence of a head-to-head clinical comparison of two therapies, an unanchored matching-adjusted indirect comparison (MAIC) was conducted by adjusting the patient characteristics using individual patient data from pivotal phase III trial of edaravone dexborneol and published aggregated data of dl-3-n-butylphthalide. Health outcomes were measured in quality-adjusted life years (QALYs). Utilities and costs (Chinese Yuan, CNY) were derived from publications and open-access database. One-way and probabilistic sensitivity analyses were performed to evaluate the robustness of results.

**Results:**

Compared with patients in dl-3-n-butylphthalide arm, edaravone dexborneol arm was found to be cost-effective in 90 days and highly cost-effective as the study horizons extended. With a similar direct medical cost, patients in edaravone dexborneol arm slightly gained an additional 0.1615 QALYs in life-time. In the long term (40 years), patients in edaravone dexborneol arm and dl-3-n-butylphthalide arm yielded 8.0351 and 7.8736 QALYs with the overall direct medical cost of CNY 29,185.23 and CNY 29,940.28, respectively. The one-way sensitivity analysis suggested that the incremental cost-effectiveness ratio was most sensitive to the price of edaravone dexborneol and dl-3-n-butylphthalide.

**Conclusion:**

Edaravone dexborneol is a cost-effective alternative compared with dl-3-n-butylphthalide for acute ischemic stroke patients in current medical setting of China.

## Introduction

Stroke poses a major health and social economic burden worldwide [1]. According to a nationwide population-based survey conducted by Wang w, et al. [2], the annual incidence of stroke in China was 345 per 100,000. Of all strokes, ischemic stroke accounts for 82% and results in over 30 billion Chinese Yuan (CNY) direct and indirect losses per-year, placing a heavy burden on society, families, and individuals [3].

Acute ischemic stroke (AIS) is the leading cause of hospitalization for neurological disorders [4]. Brain cytoprotective therapy has been recommended to treat AIS by Chinese Stroke Association guidelines for clinical management of cerebrovascular disorders in 2023 [5]. Edaravone dexborneol (ED) is a novel cytoprotective drug developed in China that comprises edaravone and (+)-borneol in a 4:1 ratio and was approved by the National Medical Products Administration (NMPA) in July 2020 for the treatment of AIS patients. Phase II and III studies of ED have demonstrated that Chinese patients treated with ED within 48 h of AIS onset have better functional outcomes than those treated with edaravone alone [6, 7]. Based on the solid evidence, ED was recommended to treat AIS patients by Chinese Stroke Association (evidence at level B, recommendation Grade IIa [5]). Other brain cytoprotective therapies were considered as Grade IIb recommendations [5]. DI-3-n-butylphthalide (NBP) is a drug developed independently in China that acts on multiple pathological links of acute ischemic stroke [8] and was approved for treatment of AIS by the NMPA in 2010. Clinical studies have demonstrated that NBP can improve the oxidative stress response of the nervous system [9], inhibit neuronal apoptosis and autophagy [10]. Currently, both of the two innovative brain cytoprotective drugs have been the most widely prescriptions for AIS in China and have been included in China Medicine Catalogue of the National Basic Medical Insurance, Industrial Injury Insurance and Maternity Insurance (2022 edition) (http://www.nhsa.gov.cn/art/2023/1/18/art105_10081.html). However, it is uncertain that which one of these two pharmaceuticals costs less and is more effective.

The purpose of this study was to evaluate the cost-effectiveness of ED versus NBP for the treatment of AIS from the Chinese healthcare system perspective.

## Materials and methods

### Model structure

This cost-effectiveness analysis comprised a short-term decision tree model of AIS (90 days), followed by a long-term Markov model with a life-time horizon. The model was implemented in Microsoft Excel 2019.

**Fig. 1:**
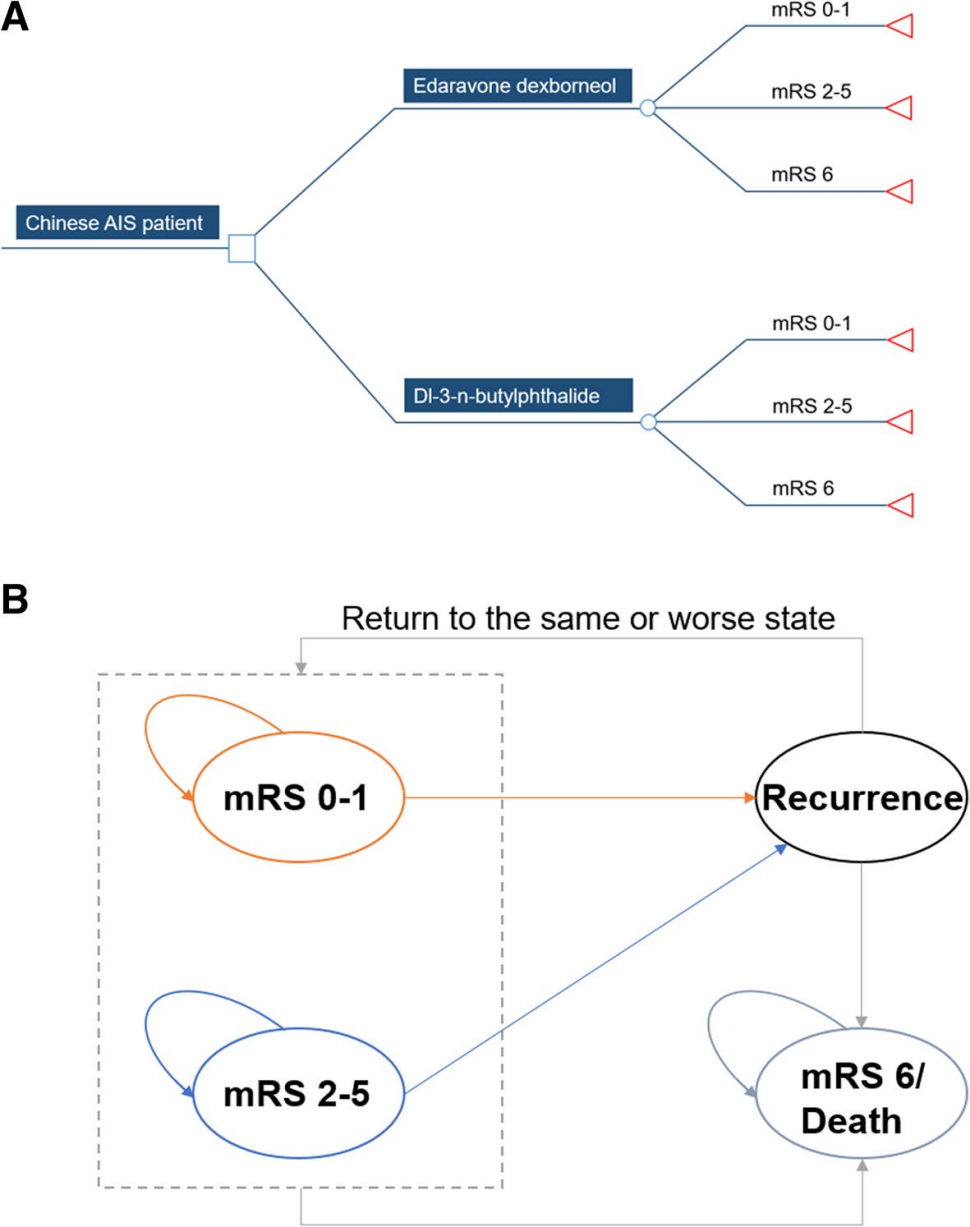
Model structure. **A** Short-term decision tree model and **B** long-term Markov model. Patients aged 60 with acute ischemic stroke within 48 h after stroke onset entered the model and received either edaravone dexborneol or Dl-3-n-butylphthalide for the first 90 days. In decision tree model, patient would distribute among different health states on day 90 and then enter Markov model. Patients may either remain in their current state, experience a recurrent ischemic stroke and transition to a state of equal or greater disability, or die from age-specific mortality or excess mortality. mRS indicates modified Rankin Scale. The health states include mRS 0–1, mRS 2–5 and mRS 6 (Death). Patients may have a stable health state, or transit to a state of equal or greater disability after recurrent stroke, or died

The short-term decision tree model (Fig. 1A) was constructed to estimate the costs and health outcomes of patients in the two treatment arms from randomization to the 90th day (D90) after treatment. According to modified Rankin Scale (mRS) score, patients were assumed to divided into three health states: no disability (mRS 0–1), with disability (mRS 2–5) and dead (mRS 6). The model assumed that patients initially entering the short-term decision tree model were in mRS 0–1 score state.

The patients mRS score health states on D90 from decision tree model was used as the initial health state of Markov model (Fig. 1B), and the first 90 days was filled up to 1 year in the 0 cycle. The cycle length was 1 year. At the end of each cycle, patient either remained in their current health state, or experienced a recurrent ischemic stroke and then transitioned to a state of equal or greater disability, or died. A half-cycle correction was applied to both costs and QALYs for all cycles except for 0 cycle which is from the decision tree model.

### Selection of studies and parameters for analysis

The absence of head-to-head trial for ED versus NBP is a challenge for this study. Matching-adjusted indirect comparison (MAIC) analyses have been reported as a new tool for timely comparative effectiveness research [11], and have previously been conducted for effectiveness comparison and cost analysis between different therapies [11–16]. This study applied unanchored MAIC to compare the effectiveness of ED versus NBP, based on the individual patient data (IPD) of the TASTE trial (ED) and aggregated data of the NBP trial below.

Clinical data of ED arm in our study was based on the TASTE trial (NCT02430350), a phase III, randomized, double-blind, parallel, comparative study for comparing the efficacy and safety of ED to edaravone alone for the treatment of AIS, enrolled 599 patients in the treatment of ED from May 2015 to December 2016 of 48 centers in China [7]. Patients in the ED arm received an edaravone dexborneol intravenous infusion of 37.5 mg/dose (edaravone, 30 mg; (+)-borneol, 7.5 mg), once every 12 h and continued for 14 days [7]. For NBP arm, a phase IV, prospective, open-label, single-arm, real world trial that enrolled 2771 patients from March 2012 to December 2014 of 74 centers in China was included [17]. The NBP arm received an dl-3-nbutylphthalide injection (25 mg/100 ml) of 100 ml twice a day and continued for 14 days [17].

All the baseline characteristics including age, gender, National Institutes of Health Stroke Scale (NIHSS) score, stroke history etc. presented in both the NBP and ED study were selected for population-adjusted indirect comparison, and the baseline characteristics in two studies before and after MAIC were shown in Table 1. MAIC was performed using the software of SAS 9.4 and adjusted proportions and risk differences were calculated using a multivariate linear regression model. The patients’ distribution of mRS score on day 90 in two studies before and after MAIC was presented in Table 2.

**Table 1:** Patient demographics and baseline characteristics before and after MAIC

Matching variable	Dl-3-n-butylphthalide	Edaravone dexborneol	
		Before MAIC	After MAIC
Effective sample size, n	2771	599	585
Mean age (SD), year	62.29 (11.54)	61.80 (9.46)	62.29
Male, n (%)	1890 (68.21%)	404 (67.45%)	68.21%
Mean intravenous medication time window (SD), hour	33.54(21.08)	28.01(10.96)	33.54
History of AIS, n (%)	791(28.55%)	174 (29.05%)	28.55%
Mean NHISS score (SD)	6.99(5.62)	7.07 (2.96)	6.99
Diagnosis with hypertension, n (%)	1871(67.52%)	390 (65.11%)	67.52%
Diagnosis with diabetes, n (%)	714 (25.77%)	151 (25.21%)	25.77%
Diagnosis with hyperlipidemia, n (%)	250(9.02%)	44(7.35%)	9.02%

*MAIC* Matching-adjusted indirect comparison, *mRS* Modified Rankin scale, *NIHSS* National Institute of Health Stroke Scale, *SD* Standard deviation, *AIS* Acute ischemic stroke

**Table 2:** Patients distribution of mRS score on day 90 before and after MAIC

mRS score	Dl-3-n-butylphthalide	Edaravone dexborneol	
		Before MAIC	After MAIC
mRS 0–1	63.80%	67.10%	67.00%
mRS 2–5	36.20%	32.90%	33.00%
mRS 6	0.00%	0.00%	0.00%

*MAIC* Matching-adjusted indirect comparison, *mRS* Modified Rankin scale

### Transition probabilities

Excess death rate due to stroke was incorporated into the long-term Markov model as the hazard ratio for each mRS health state by using the age-specific death rate multiplied by the hazard ratio for each mRS health state [18]. The stroke death hazard ratio of mRS 0–1 and mRS 2–5 contracted from the published study by Boudreau et al. [19]. The age-specific natural mortality rate was drawn from and adjusted according to the causes of death in 2020 reported in the China Population and Employment Statistical Yearbook 2021 [20].The probability of transferring to death after recurrent stroke was obtained from publication [21] and we assumed patients in mRS 0–1 state would transfer to same status (mRS 0–1) or worse status (mRS 2–5, death) after recurrent stroke. Sources and values of all transition probabilities and mortality rate were shown in Table 3.

**Table 3:** Model input parameters

Model input	Base case	Range	Distribution	Reference
Stroke recurrent rate				
Recurrent rate (0–1 year)	5.90%	-	-	[22]
Recurrent rate (1–2 year)	3.60%	-	-	[22]
Recurrent rate (2–3 year)	2.50%	-	-	[22]
Recurrent rate (3–4 year)	2.20%	-	-	[22]
Recurrent rate (4–5 year)	2.20%	-	-	[22]
Recurrent rate (5–6 year)	2.70%	-	-	[22]
Recurrent rate (6–7 year)	2.70%	-	-	[22]
Recurrent rate (7–8 year)	2.30%	-	-	[22]
Recurrent rate (8–9 year)	2.80%	-	-	[22]
Recurrent rate (after 9 year)	1.60%	-	-	[22]
**Death hazard ratios**				
mRS 0–1	1.00	(1.00-1.20)	lognormal	[19]
mRS 2–5	2.50	(1.70–3.80)	lognormal	[19]
**Natural mortality rate (per year)**				
60–64 years	0.75%	-	-	[20]
65-69years	1.17%	-	-	[20]
70–74 years	2.03%	-	-	[20]
75–79 years	3.56%	-	-	[20]
80–84 years	6.29%	-	-	[20]
85–89 years	10.28%	-	-	[20]
≥ 90 years	16.17%	-	-	[20]
**Costs (2021, Chinese Yuan)**				
Edaravone dexborneol /piece	33.00	33.00-48.80	-	The price from the National Basic Medical Insurance, Industrial Injury Insurance and Maternity Insurance Drug Catalogue (2022 edition)
Dl-3-n-butylphthalide/ bottle	116.76	116.76–139.00	-	
Hospitalization costs for mRS(0–1)	12613.70	12428.04-12801.87	gamma	[23]
Hospitalization costs for mRS(2–5)	17222.62	16845.03-17606.49	gamma	[23]
Hospitalization costs for mRS(6)	13950.96	12819.43-15155.26	gamma	[23]
Rehabilitation and secondary prevention costs for mRS(0–1)	9264.26	8976.99-9557.81	gamma	[23]
Rehabilitation and secondary prevention costs for mRS(2–5)	14238.24	13460.47-15048.63	gamma	[23]
**Mean length of hospitalization**	9.90	-	-	[24]
**Health Utility**				
mRS 0–1	0.84	0.66–0.92	beta	[19]
mRS 2–5	0.47	0.24–0.66	beta	[19]
Disutility of recurrent stroke	0.09	0.06–0.11	beta	[25]
**Discount rate**	5%	0-8%	-	[26]
**Transition probability of recurrent stroke**				
mRS 0–1 to mRS 0–1	39.50%	-	-	Assumption
mRS 0–1 to mRS 2–5	39.50%	-	-	Assumption
mRS 0–1 to mRS 6	21.01%	-	-	[21]
mRS 2–5 to mRS 2–5	78.99%	-	-	Assumption
mRS 2–5 to mRS 6	21.01%	-	-	[21]

*mRS* Modified Rankin scale

### Cost

The direct medical costs were considered in this analysis, including 14-days standard treatment costs of ED and NBP, other drug costs, hospitalization costs by mRS score, rehabilitation costs and post-stroke costs for secondary prevention (15 days to 90 days). The price of ED and NBP was obtained from Medicine Catalogue of the National Basic Medical Insurance, Industrial Injury Insurance and Maternity Insurance (2022 edition). The 14-days treatment cost of ED and NBP were calculated as daily treatment cost multiplied by 14 days. The average length of hospitalization was based on the China Healthcare Statistical Yearbook 2021 [24]. Hospitalization costs and post-stroke costs for secondary prevention by mRS score were extracted from the China National Stroke Registry (CNSR) [27] and published study by Pan Y, et al. [23]. The CNSR database covered 132 hospitals and included 21,902 stroke patients in China [27]. We assumed that the hospitalization costs and post-stroke costs for secondary prevention of mRS 2–5 were equal to the costs of health state of mRS 3–5 based on the study by Pan Y, et al. [23]. All costs were converted to 2021 CNY by the medical care component of China’s Consumer Price Index (CPI) (Table 3). Costs were discounted at an annual rate of 5% according to China guidelines for pharmacoeconomic evaluations (2020) [26].

### Utility

Utilities of health state were derived from the published study conducting in US population by Boudreau et al. [19] due to the lack of relevant Chinese study, and were discounted at an annual rate of 5% (Table 3). The disutility of stroke attack was considered in our study, and we assumed the annual disutility referenced from the published study by Mok CH, et al. [25].

### Cost-effectiveness analysis

Incremental cost-effectiveness ratio (ICER) was used to indicate cost-effectiveness which was calculated by dividing the incremental cost by the incremental quality-adjusted life year (QALY) gained, and a willingness-to-pay (WTP) threshold of 1–3 times of Chinese gross domestic product (GDP) per capita was applied to determine whether the more effective intervention is worthy of the extra money spent (Chinese GDP per capital = CNY 80,976, 3 times of Chinese GDP per capital = CNY 242,928 in 2021) as recommended by the China guidelines for pharmacoeconomic evaluations (2020) [26].

### Sensitivity analyses

One-way deterministic sensitivity analyses and probabilistic sensitivity analyses (PSA) were both performed to evaluate the sensitivity of the model to uncertainty in model parameters (Table 3). The one-way sensitivity analysis was undertaken to identify variables that significantly influence the ICER, and presented by a tornado diagram. The PSA was performed by using a Monte Carlo simulation (1,000 iterations) with parameter inputs sampled from the fixed distributions (Table 3), which presented in a scatter plot of incremental QALY and incremental cost and a cost-effectiveness acceptability curve.

## Results

### Baseline demographics

Before MAIC, baseline characteristics including age, gender, NIHSS score and history of diagnosed disease (AIS, hypertension, diabetes and hyperlipidemia) were similar between the NBP arm and the ED arm. Patients who received arterial or venous thrombolytic drugs after stoke onset were excluded in the phase 3 trial of ED. The information of recanalization therapy were not reported in the phase 4 trial of NBP. 585 out of 599 patients in the ED arm were finally included in the efficacy analysis, therefore, after MAIC, only 585 patients for efficacy analysis could be included in the ED arm. Finally, a total of 585 patients in ED arm [7] and 2771 patients in NBP arm [17] were included in the unanchored MAIC analysis for effectiveness comparison. The baseline demographic and disease characteristics before and after MAIC were presented in Table 1 and all the available variables after MAIC in two arms were comparable.

### Base-case analysis

After MAIC, patients in the ED arm had higher proportion (67.00% vs. 63.80%) of the state of no disability (mRS 0–1) and lower proportion (33.00% vs. 36.20%) of the state with disability (mRS 2–5) than those in the NBP arm (Table 2). Table 4 shows the base-case analysis results of day 90, 5 years and life-time cost-effectiveness analysis. Within a life-time horizon, the overall direct medical cost in ED arm was CNY 29185.20, while the NBP arm was CNY 29940.3, and the effectiveness of ED arm and NBP arm was 8.0351 QALYs and 7.8736 QALYs, respectively. Compared with NBP, ED resulted in cost-savings of CNY 755.05 and gained an additional 0.1615 QALYs in life-time, demonstrating that treatment with ED was dominant to treatment with NBP.

**Table 4:** Results of the base-case analysis

Time horizon		Edaravone dexborneol	Dl-3-n-butylphthalide	Difference
Day 90	Cost (CNY)	19302.20	19979.61	-677.41
	QALY	0.1708	0.1694	0.0014
	ICER			Dominant
5 years	Cost (CNY)	24957.11	25704.09	-746.98
	QALY	2.7230	2.6780	0.0450
	ICER			Dominant
Life-time (40 years)	Cost (CNY)	29185.23	29940.28	-755.05
	QALY	8.0351	7.8736	0.1615
	ICER			Dominant

*CNY* Chinese Yuan, *QALY* Quality-adjusted life years, *ICER* Incremental cost-effectiveness ratio

### One-way and probabilistic sensitivity analyses

**Fig. 2:**
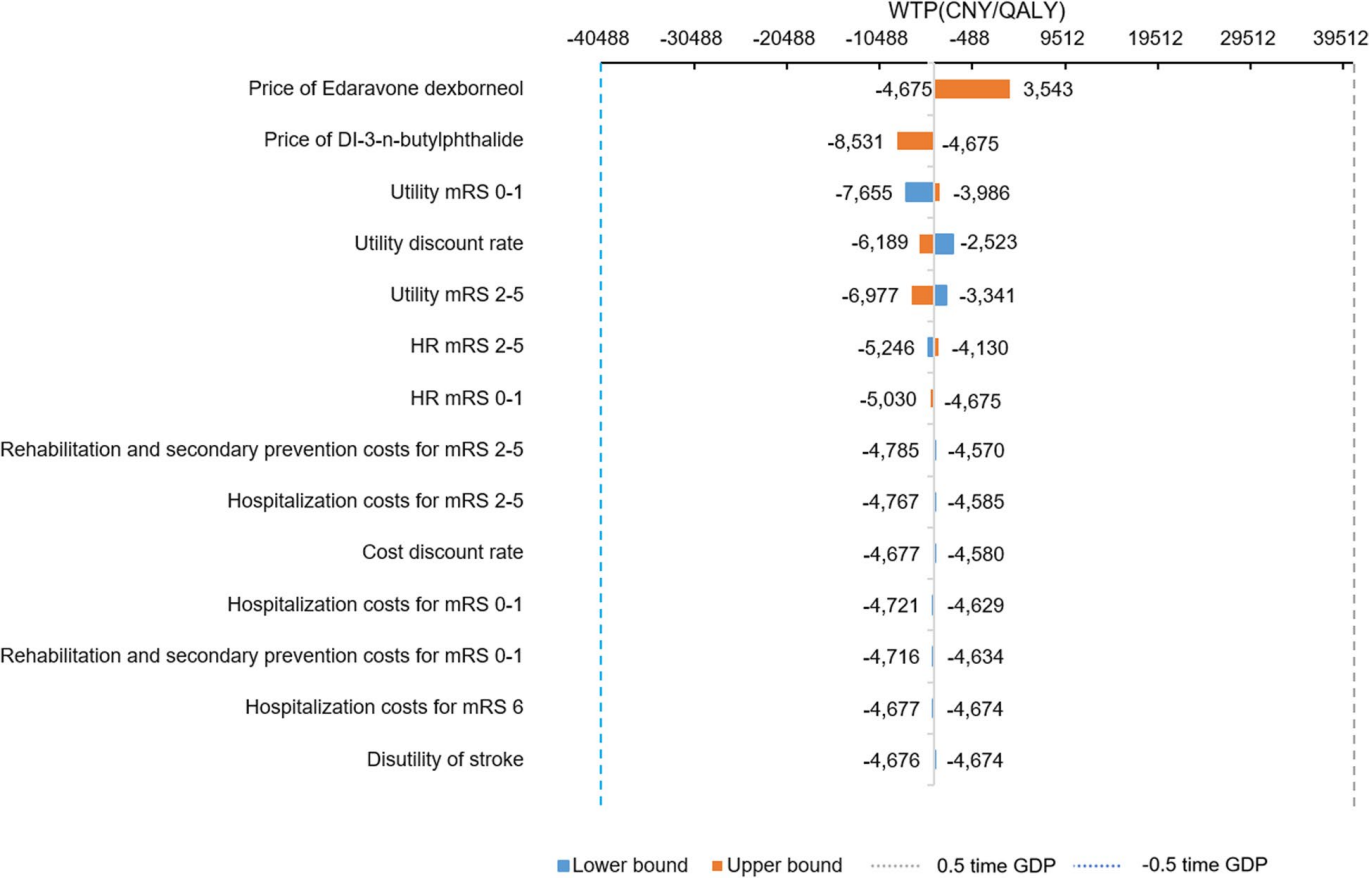
Tornado diagram. *mRS* Modified Rankin Scale, *HR* Hazard ratio

 The tornado diagram in Fig. 2 showed the impact of parameter variation on ICER. The most significant driver of ICER was the price of ED, followed by the price of NBP and utility of mRS (0–1).

**Fig. 3:**
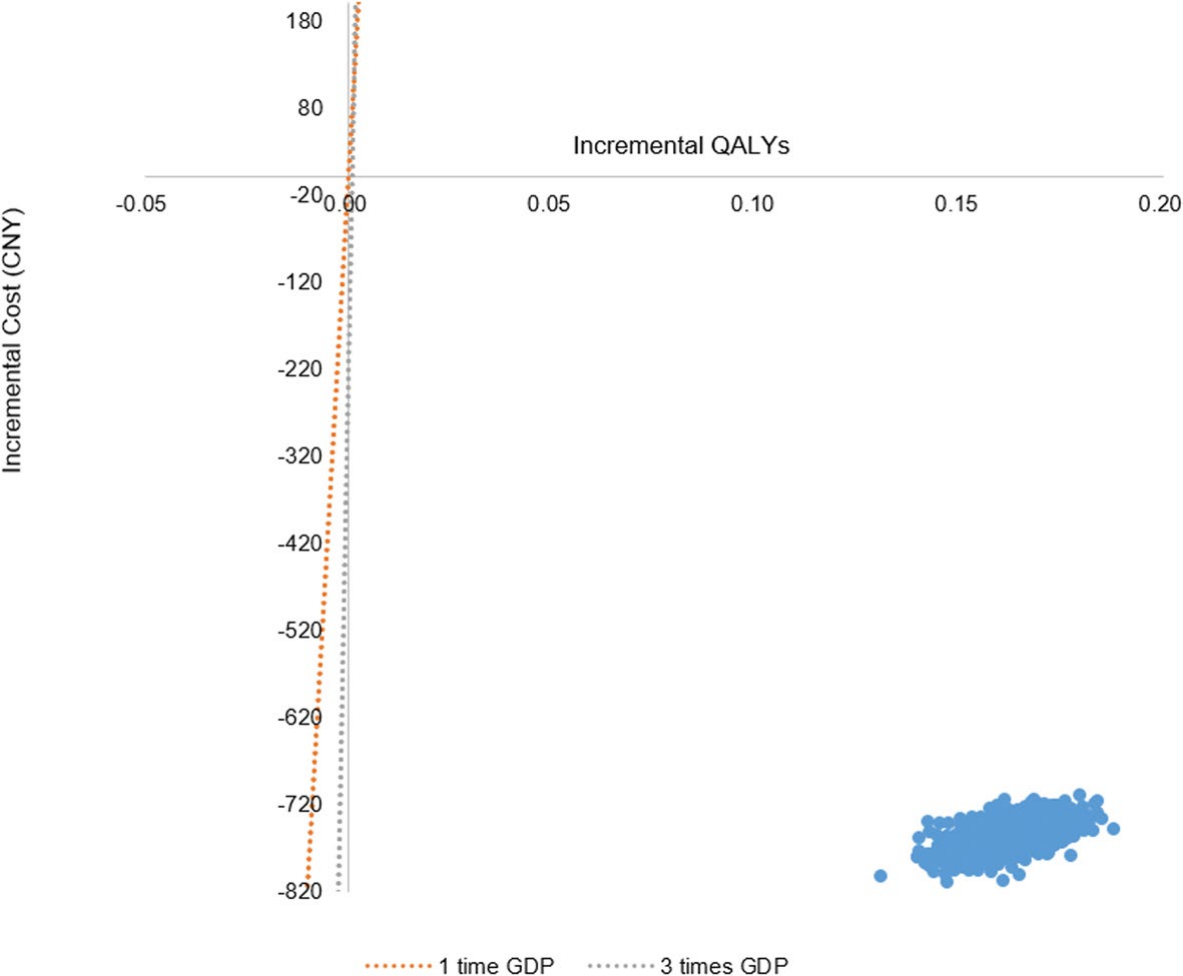
ICER scatter plot. *ICER* Incremental cost-effectiveness ratio, *QALYs* Quality-adjusted life years, *CNY* Chinese Yuan

**Fig. 4:**
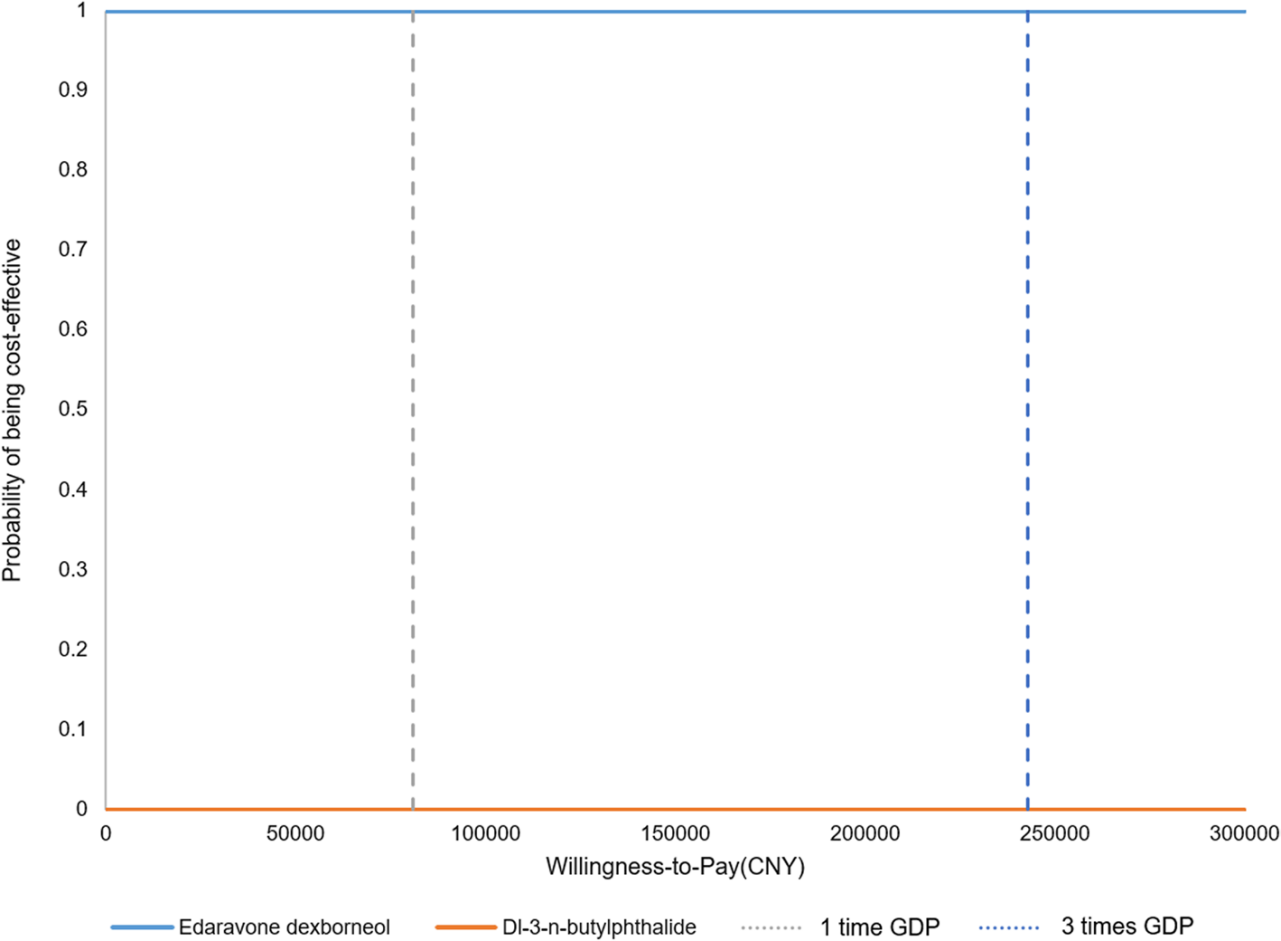
Cost-effectiveness acceptability curve. *WTP* Willingness-to-pay, *QALY* Quality-adjusted life year

 The PSA results were shown in Figs. 3 and 4. The scatter plot of incremental QALY and incremental cost is shown in Fig. 3. With 1000 iterations, ED treatment was 100% cost-effective at a WTP threshold of 80,976 (Chinese GDP per capital in 2021) CNY/QALY while NBP treatment had 0% probability to be the cost-effective strategy at a WTP threshold of 80,976 CNY/QALY. The cost-effectiveness acceptability curve of both treatments is shown in Fig. 4.

## Discussion

This is the first study to evaluate the cost-effectiveness of ED versus NBP in the treatment of AIS from the perspective of Chinese healthcare system. A model combining a decision tree and a Markov model were applied to evaluate both short and long-term cost-effectiveness of ED versus NBP in China. The main findings indicated that ED was a dominant cost-saving strategy with lower cost and greater QALY gained compared with NBP under a WTP threshold range of 1–3 times GDP per capital. Probabilistic sensitivity analysis with bootstrapping method indicating that, the probability of ED being cost-effective compared with NBP is 100% under a 1–3 times GDP threshold. These findings can provide evidence for clinical and economic decisions in managing patients with AIS.

In the absence of a head-to-head clinical study, indirect comparison of treatments can be conducted via rigorous methodology that adjusts for inherent cross-study differences, aiming to reduce biases [11]. The unanchored MAIC analysis was applied in this study to match individual patient data from TASTE trial of ED arm (provided by Simcere Pharmaceutical Co., Ltd) with published aggregated data of NBP arm [7, 17] to minimize the impact of baseline characteristics of the two different studies and to reach a comparable clinical effectiveness which was the patients distribution of mRS scores on D90 after stroke onset.

According to the one-way sensitivity analysis, the top factor with great influence on ICER was the price of ED, followed by the price of NBP, which were different from the findings of cost-effectiveness of ED versus edaravone for the treatment of AIS in China by Shi FH, et al. [18]. The price of ED was lower, the treatment of ED was more cost-effective. In the study, the primary reason which was resulted in the difference of total direct medical costs was attributed to unequal 14-days treatment costs. The daily treatment cost of NBP and ED are 233.52 CNY and 198 CNY respectively. Additionally, the hospitalization costs, rehabilitation costs and post-stroke costs for secondary prevention related to difference mRS score. Thus, different proportion of mRS patients on day 90 in the two original trials also led to inconsistent total direct medical costs. We also found that the cost-effectiveness domination of the ED therapy is increasing over study time, which was similar to the finding of cost-effectiveness of ED versus edaravone for the treatment of AIS in China by Shi FH, et al. [18].

However, there are some limitations in this study. From the perspective of study design, the phase III clinical trial of ED was performed from May 2015 to December 2016 among 48 centers in China while the phase IV real world study of NBP was performed from March 2012 to December 2014 among 74 centers in China, they were different in study time and study design [7, 17] such as the inconsistent patients inclusion and exclusion criteria and treatment regimen. During the different study period, an updated Chinese guidelines for the diagnosis and treatment of AIS (edition 2014) had new recommendations on the control of blood pressure, thrombolytic therapy, early rehabilitation and secondary prevention compared with the Chinese guidelines for the diagnosis and treatment of AIS (edition 2010). Thus, the above updates of treatments might have different impacts on patients in two clinical trials. But the risk factors were not observed and collected in our study. It was a limitation of our study. Using MAIC method could adjust the observed differences in patients’ demographic and disease characteristics reported in studies. Although the study has included the most important prognostic factors, which are only those can be observed and reported, it is not possible to include all prognostic factors and effect modifying factors. MAIC analysis cannot replace the robustness of randomized clinical trial (RCT) results, and further validation of RCT is still needed in the future. Besides, 99.68% of the patients in NBP arm had concomitant medication [17], while the patients in ED arm strictly followed the monotherapy rules [7]. Therefore, the effectiveness of NBP might be overestimated in MAIC. Those differences and limitations could only be avoided with well-controlled head-to-head clinical study [28, 29]. Nonetheless, while the MAIC analysis cannot replace head-to-head RCT, it might still be the best choice to provide references for the comparative effectiveness of drugs in the absence of head-to-head RCT [11]. Additionally, the health utility data should prioritize from Chinese target study population.However, in the absence of health utility data for Chinese stroke patients after retrieving domestic and foreign publications, the utility data in our analysis were derived from literature studies, which are those from United States. Although this method has been used widely among published stoke studies, it would inevitably lead to uncertainty when compared to the real clinical settings in China. It is a limitation of the present study. In order to test the impact of above parameters variation on the results, one-way sensitivity analysis and probabilistic sensitivity analysis were performed. Overall, these analyses demonstrated that the model results are robust. Finally, the proportion of patients with mRS 0–1 on day 90 was 67.0% in the phase 3 clinical trial of ED. Of course we know the RCT data is not completely equivalent to the proportion in the real world clinical practice. ED was approved for treatment of AIS by the NMPA in 2020 in China. Up to now, there is no high-quality real world data study available. Thus, the probability of patients with mRS 0–1 at day 90 for ED therapy in the real world clinical practice was unknown. It was a limitation of our data sources. Before obtaining better real world data, the cost-effectiveness analysis based on clinical trials still have clinical guidance for patients and clinicians. Further analysis with real world data should be conducted in the future.

## Conclusion

This study demonstrated that ED was a cost-effective alternative to treat AIS compared with NBP in China. Additionally, our study could provide the evidence to decision makers for selecting the high value intervention in the China DRG/DIP payment system.

## Data Availability

The datasets used and/or analyzed during the current study are available from the corresponding author on reasonable request.

## Abbreviations

AIS: Acute ischemic stroke
CNY: Chinese Yuan
CNSR: China National Stroke Registry
CPI: China’s Consumer Price Index
D90: 90th day
ED: Edaravone dexborneol
GDP: Chinese gross domestic product
ICER: Incremental cost-effectiveness ratio
IPD: Individual patient data
MAIC: Matching-adjusted indirect comparison
mRS: Modified Rankin Scale
NBP: Dl-3-n-butylphthalide
NMPA: National Medical Products Administration
NIHSS: National Institutes of Health Stroke Scale
PSA: Probabilistic sensitivity analyses
QALYs: Quality-adjusted life years
WTP: Willingness-to-pay
RCT: Randomized clinical trial
